# Evaluating bacterial growth in raw, frozen, and heat-treated colostrum inoculated with fecal *Escherichia coli*

**DOI:** 10.3168/jdsc.2025-0756

**Published:** 2025-06-18

**Authors:** A.M. McKane, T.A. Westhoff, S. Klaessig, C. Altier, K.E. Bell, P.D. Pavinski Bitar, S. Mann

**Affiliations:** Department of Population Medicine and Diagnostic Services, College of Veterinary Medicine, Cornell University, Ithaca, NY 14853

## Abstract

Providing newborn calves with sufficient high-quality colostrum is a critical calf-management strategy to support health, survival, and future productivity. Unfortunately, colostrum may also serve as a fomite for disease when contaminated with bacteria at harvest, during storage, or during reheating before feeding. Thermal processing, including heat treatment (HT; 60°C for 60 min) and freezing (FR; −20°C), are common strategies to manage bacterial contamination. Although both strategies maintain IgG concentrations, they destroy colostral immune cells, and HT is known to decrease the concentration and activity of certain bioactive factors such as complement and IgA. We hypothesized that HT and FR would influence the inherent antibacterial properties of bovine colostrum compared with unprocessed, refrigerated colostrum (RW). Our objective was thus to compare growth of *Escherichia coli* in RW, HT, and FR bovine colostrum. Sterile colostrum samples were collected from Holstein cows (n = 11) on a commercial dairy in New York State and divided into 4 aliquots. One aliquot was submitted for bacterial culture to exclude samples with any bacterial growth (n = 4). The remaining 7 samples were used for the experiment. Aliquots were either RW (4°C for 20 h; n = 7), HT (60°C for 60 min, then 4°C for 19 h; n = 7), or FR (−20°C for 20 h; n = 7). Immediately before inoculation of samples for a bacterial kinetics assay, a dried bovine colostrum-based replacer (CR; Ultra Start 150, Sav-A-Calf, Chilton, WI) was prepared from a single bag according to package instructions as a nutrient-rich control (n = 7). The prepared CR aliquots underwent bacterial culture to ascertain the absence of bacterial growth before inoculation. To simulate contamination that may occur on-farm, all samples were inoculated with 10^4^ cfu/mL *E. coli* (WM1; 060913 P0lA, isolated from bovine feces) and tested in a bacterial kinetics assay at 37°C. At 0, 2, 4, 6, 8, and 24 h, growth of *E. coli* was quantified on MacConkey agar plates inoculated at 37°C. Relative to colostrum replacer, growth of *E. coli* was lower in RW and FR colostrum from 4 to 24 h and lower in HT colostrum at 6 and 8 h. These results demonstrate inherent microbial growth-inhibiting activity in RW colostrum and suggest that FR better preserved this activity than HT. Our study contributes to understanding the effect of thermal processing on antimicrobial properties of colostrum with the goal of helping to inform colostrum-management strategies for dairy producers. Specifically, our data emphasizes the need to practice hygiene during all steps of the colostrum harvest and storage process, including after completion of HT, because this renders colostrum more susceptible to bacterial growth. Similarly, the cooling and heating steps of stored colostrum should be rapid to minimize growth of *E. coli*.

## Highlights

• Raw colostrum had growth-inhibiting activity against a fecal strain of *E. coli*.
• Freezing colostrum preserved its growth-inhibiting activity.
• Heat treatment (60°C for 60 min) reduced colostrum's growth-inhibiting activity.

## Graphical Abstract

**Figure:**
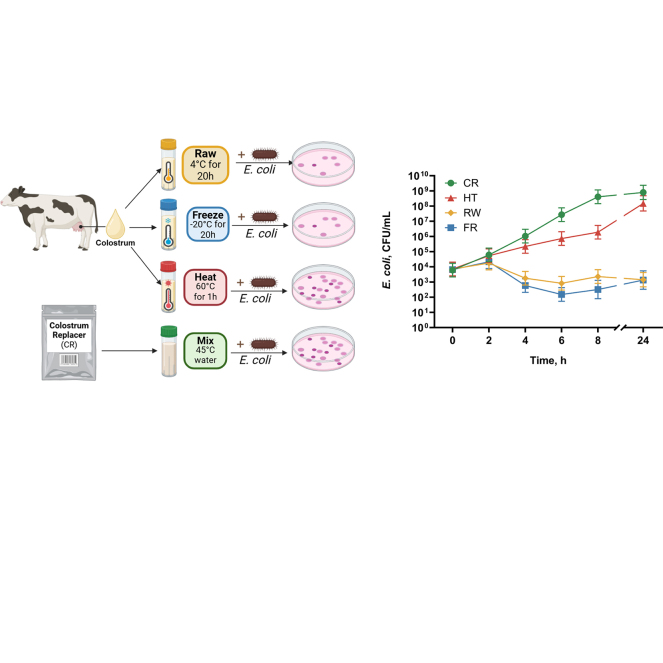
**Summary:** This project evaluated the effect of postharvest thermal processing on the growth-promoting or -inhibiting activity of bovine colostrum. Colostrum was collected aseptically from Holstein cattle (n = 7), and aliquots were submitted to 3 treatments: cooling at 4°C only (RW), freezing at -20°C (FR), or heat treatment at 60°C for 60 minutes (HT). Colostrum replacer (CR) was included as a control. The day after treatments were applied, each sample was inoculated with a bovine gastrointestinal strain of *Escherichia coli* and incubated together at 37°C for 24 hours. Growth of *E. coli* was quantified on MacConkey agar plates immediately after inoculation (0 hours) as well as at 2, 4, 6, 8, and 24 hours. The resulting growth curves showed that cooled and freeze-thawed colostrum inhibited growth of *E. coli*, whereas heat-treated colostrum and colostrum replacer did not. These findings show that HT reduced the inherent antibacterial activity of colostrum, highlighting that colostrummanagement practices have important implications for bacterial growth.

Providing sufficient high-quality colostrum to calves within the first few hours of life is widely accepted as a hallmark of successful colostrum-management programs (Godden, 2008; Hammon et al., 2020). Aside from its high nutritional value, colostrum also contains high concentrations of bioactive factors including Ig, growth factors, hormones, cytokines, and antimicrobial substances (Godden, 2008; Tortadès et al., 2023). These factors aid in achieving transfer of passive immunity, which provides immunity for agammaglobulinemic bovine neonates (Lombard et al., 2020).

Producers may choose to process colostrum before storage to (1) reduce microbial load and (2) preserve immunologically important components. Heat treatment (**HT**) is a commonly used postharvest processing method that lowers the microbial load. This is important to achieve transfer of passive immunity because bacterial contamination reduces absorption of Ig (James et al., 1981). Additionally, HT has been used to decrease the risk of pathogen exposure to calves, including *Mycoplasma bovis*, *Escherichia coli*, and *Salmonella enteritidis* (Godden et al., 2006). The 60°C for 60 min HT protocol, also known as low temperature, long time (**LTLT**), is preferred to higher temperature methods because it remains effective at killing bacteria while preserving most maternal IgG molecules (Elizondo-Salazar et al., 2010; Rabaza et al., 2023). Although the use of heat to destroy colostral microbial contaminants has a long and well-documented history (Elizondo-Salazar et al., 2010; Wörmann et al., 2023), the effect of thermal processing on other bioactive factors has only recently been the topic of investigation. Notably, we have recently shown that HT killed colostral immune cells, and decreased concentrations of IgA and IGF-1, as well as protein abundance and activity of the complement pathway (Mann et al., 2020; Chandler et al., 2023). These factors are important for gastrointestinal development and local gut immunity in bovine neonates (Ontsouka et al., 2016; Ghaffari et al., 2021), but may also play a role in determining antimicrobial properties of bovine colostrum.

Freezing (**FR**) colostrum is also commonly used as a preservation method, at a typical temperature of −20°C. Regarding the first goal of processing colostrum (reducing microbial load), FR differs from HT in that microbial load is not typically reduced (Stewart et al., 2005), but further growth is halted. Compared with HT, the effect of FR on immunologically important components is only beginning to receive interest. Some authors have examined this question indirectly by measuring the outcome of feeding freeze-thawed colostrum to calves. Calves fed colostrum stored frozen for 24 h had similar concentrations of serum IgG compared with those fed fresh colostrum (Holloway et al., 2001). In another study, lactoferrin, an antimicrobial protein in colostrum, had similar or higher concentrations in the serum of calves fed fresh or freeze-thawed colostrum in the first week of life (Holloway et al., 2002). It has also been shown that quick FR of colostrum at −80°C lyses colostral leukocytes (Donovan et al., 2007), a finding that was repeated in colostrum samples frozen at −20°C overnight (Chandler et al., 2023), whereas complement pathway activity was maintained at a level similar to that of raw colostrum (Chandler et al., 2023).

The net influence of these thermal processes on the overall inherent antibacterial properties of thermally processed bovine colostrum is not yet fully understood. As such, our objective was to compare the growth of an *E. coli* strain previously isolated from bovine feces (i.e., a possible environmental contaminant) in unprocessed, refrigerated only (**RW**), HT, and FR colostrum. We hypothesized that HT and FR would influence the inherent antibacterial properties of bovine colostrum.

Colostrum samples were collected from clinically healthy Holstein cows (n = 11) at the morning milking (0930 h) between June and July 2023 at a commercial dairy farm in New York State. Samples were collected directly from a single teat in an aseptic manner into a 120-mL sterile plastic vial and immediately placed on ice. Brix % was determined in samples at room temperature using a digital refractometer (Palm Abbe, Misco, Cleveland, OH), and samples were subsequently divided into 4 aliquots of 6 mL each. Aliquots were subjected to 1 of 3 treatments (Figure 1): (1) colostrum was cooled and stored at 4°C (RW), (2) colostrum was frozen at −20°C overnight (FR), and (3) colostrum was immersed in a 60°C water bath, removed after 60 min and stored at 4°C until the next morning (HT). On the morning of the bacterial kinetics assay (the day after colostrum was collected and treated), dried bovine colostrum-based replacer powder was prepared according to package instructions (**CR**; Ultra Start 150, Sav-A-Calf, Chilton, WI; n = 7) and included as a similarly nutrient-dense control to maternal colostrum. The fourth aliquots of each maternal colostrum and an aliquot of the dried bovine CR were submitted to the Milk Production Services (QMPS, Ithaca, NY) laboratory of the New York State Animal Health Diagnostic Center (**AHDC**) to confirm absence of any bacterial growth in standard aerobic milk culture.

**Figure 1:**
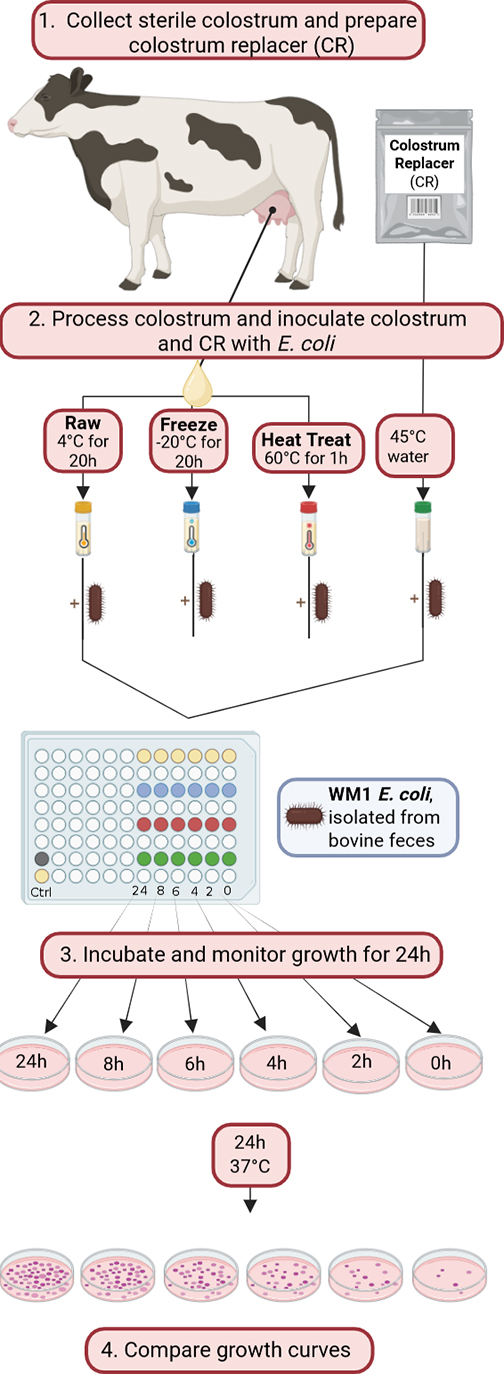
Colostrum samples (n = 7) were collected aseptically directly from a single teat of Holstein cows and subjected to 3 postharvest treatment groups: (1) heat treatment (HT), (2) freezing (FR), or (3) cooling only (RW). The next morning, colostrum replacer (CR) was prepared according to package instructions. Both colostrum and CR were plated into a 96-well plate with 1 well for each time point in the growth curve (1 well each for 0-, 2-, 4-, 6-, 8-, and 24-h time points). Each well was inoculated with 10^4^ cfu/mL of WM1 *Escherichia coli*. Uninoculated additional control wells of RW colostrum and CR were included. At each time point, a sample was drawn up from the well, appropriately diluted with PBS, and spread on a MacConkey Agar plate. At the 24-h time point, undiluted control colostrum and CR were plated as well. These plates were incubated at 37°C overnight, and then colonies were counted.

To evaluate antibacterial activity of each sample type (HT, RW, FR, CR), bacterial growth was measured over time and growth curves were calculated. The *E. coli* isolate used in the growth curves, WM1 060913 P0lA *E. coli* (WM1), was selected because it had been previously isolated from a bovine fecal sample (Van Vleck Pereira et al., 2014) and thus represented a possible environmental contaminant of colostrum. On the same day colostrum samples were collected and processed, an overnight culture of *E. coli* was prepared in Luria-Bertani (**LB**) broth. The next morning, this culture was used to inoculate fresh LB broth in a manner that was slightly modified from a previously described protocol (Sezonov et al., 2007). In brief, the overnight culture was diluted to 1:100 of the original concentration in 5 mL of fresh medium and grown at 37°C in a shaking incubator until mid-log, defined as growth after a 2-h lag period with optical density at 600 nm (**OD_600_**) values measuring between 1.0 and 1.3. The bacterial concentration (cfu/mL) of the mid-log culture was then calculated based on the OD_600_ value and subsequently diluted to 2 × 10^5^ cfu/mL with sterile PBS. This served as the starting inoculum for the growth curves in colostrum samples.

While the *E. coli* grew to mid-log, the processed colostrum and CR were diluted to 1:2 of the original concentration with sterile PBS to reduce viscosity and aliquoted into sterile 96-well plates (237.5 µL/well), which served as the vessels for the growth curves. The plates were incubated at 37°C for the duration of the experiment (24 h). Each treatment group (HT, FR, RW, and CR) had 6 separate wells (Figure 1), 1 for each time point of the growth curve (0, 2, 4, 6, 8, and 24 h). Two additional wells were plated: 1 with RW colostrum and the other with colostrum replacer, to serve as controls for contamination that may occur during the experimental process. Except for these controls, each well was inoculated with 12.5 μL of *E. coli* to achieve a starting concentration of 10^4^ cfu/mL. Immediately after inoculation, a 0-h sample was collected for each treatment group and plated on MacConkey agar. To ensure a countable number of colonies would grow on the plate, serial dilutions were carried out based on results of prior growth curves of WM1. Serial dilutions and plating were repeated after 2, 4, 6, 8, and 24 h of incubation at 37°C. The 0-, 2-, 4-, 6-, and 8-h plates were incubated at 37°C until the 24-h sample was collected (16–24 h, depending on the plate). The negative controls were plated at the same time as the 24-h treatment time point and all plates were incubated 24 h before assessing colonies.

The sample size was determined in GPower v. 3.1.9.7 (Faul et al., 2009) based on the expected difference between the concentration of *E. coli* in at least 2 groups. Assuming a mean concentration difference of 1 × 10^6^ cfu/mL with SD of 7 × 10^5^ cfu/mL and controlling for 5% type I error and a desired power of 95%, the effect size was 1.4, with a resulting sample size of 9. We increased our sample size by 20% to 11 samples to account for potential bacterial contamination. Data were analyzed in JMP v. 16.0 (SAS Institute Inc.) using repeated-measures ANOVA with the fixed effects of treatment, time, and treatment × time interaction, repeated effect of time, and a random effect of experiment date. The model assumptions of normality and homoscedasticity of the residuals were visually assessed. As a result, *E. coli* growth data were transformed using the natural logarithm to meet model assumptions. Tukey's post hoc test was used to adjust for multiple comparisons when applicable. In addition, the initial concentration of *E. coli* in each treatment was compared with subsequent time points using Dunnett's test to control for multiple comparisons. Data are reported as LSM and 95% CI. Significance was declared at *P* ≤ 0.05.

A total of 11 colostrum samples were collected for this experiment, with Brix % ranging from 15.5% to 31.2% (mean = 23.7%). Data from 4 samples had to be excluded due to contamination either on the negative control (n = 3) or bacterial growth shown in the aliquot submitted to the AHDC (n = 1); thus, the results from 7 samples were used in our statistical analysis. The uninoculated CR had no detectable growth.

In this study, we investigated differences in *E. coli* growth characteristics in the various treatment groups, which is shown in Figure 2. The number of *E. coli* at 0 h and 2 h did not differ between treatments. At 4, 6, 8, and 24 h, *E. coli* concentration was lower in RW and FR colostrum compared with HT colostrum and CR (*P* < 0.01). The concentration of *E. coli* was lower in HT colostrum compared with CR at 6 and 8 h (*P* < 0.01). Furthermore, we investigated differences in growth characteristics within each treatment over time. Compared with their respective bacterial counts at 0 h, *E. coli* counts were greater at all other time points in CR (*P* ≤ 0.01) and HT (*P* ≤ 0.02), indicating that these treatments permitted bacterial growth. Compared with 0 h, the concentration of *E. coli* in FR did not differ at 2 or 24 h (*P* ≥ 0.40), and was lower at the 4-, 6-, and 8-h time points (*P* < 0.01). Compared with 0 h in RW, *E. coli* concentration was lower at 6 h (*P* < 0.01), but did not differ at 2, 4, 8, or 24 h (*P* ≥ 0.12). Thus, *E. coli* showed no growth in both FR and RW colostrum over the 24-h period.

**Figure 2:**
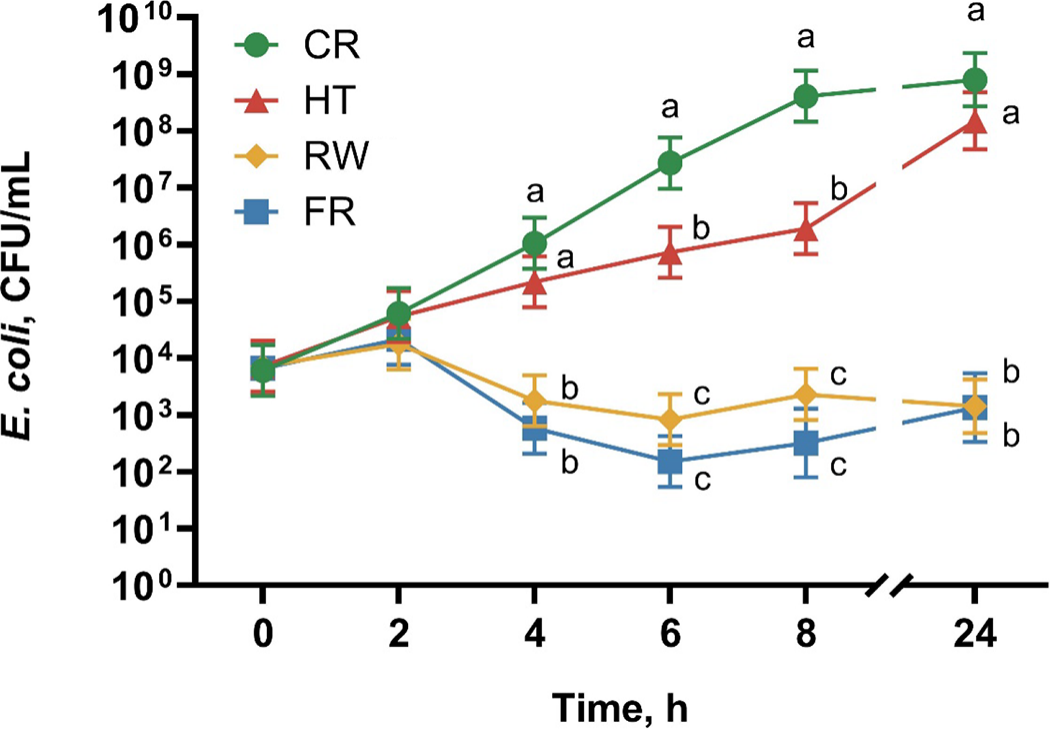
Differences in *Escherichia coli* growth over 24 h among the different treatment groups. Colostrum samples (n = 7) and colostrum replacer (CR, n = 7) were inoculated with 10^4^ cfu/mL of WM1 *E. coli* and incubated at 37°C for 24 h. Each colostrum sample was subjected to 3 postharvest treatment groups (1) heat treatment at 60°C for 60 min (HT), (2) freezing overnight at −20°C (FR), or (3) cooling at 4°C only (RW). Data were analyzed using repeated measures ANOVA with the fixed effects of treatment, time, and treatment × time, and the random effect of experiment date; results shown as LSM and 95% CI. Treatments with different lowercase letters at individual time points differ (*P* < 0.05; Tukey's test).

Prior work has shown that FR and HT influenced the macronutrient composition of colostrum (Fischer et al., 2018), physical properties such as viscosity (Rabaza et al., 2023), and concentration and activity of immunologically active components (Mann et al., 2020; Chandler et al., 2023). Our data support previous reports of inherent antibacterial activity of colostrum (Chae et al., 2017; Gao et al., 2021). In the current study, HT reduced antibacterial activity compared with RW and FR, ultimately supporting a similar bacterial load as the CR after 24 h of incubation. This result supports that at least some of the key antimicrobial components of bovine colostrum are heat labile, mirroring human colostrum (Hernandez et al., 1979).

Fresh colostrum contains high numbers of maternal leukocytes capable of phagocytosis and cytotoxicity (Newby et al., 1982; Chandler et al., 2023). However, because HT, FR, and freeze-drying as found in CR render colostral immune cells nonviable (Natan et al., 2009; Chandler et al., 2023), their role in providing antimicrobial properties does not explain the observed differences in our work and does not explain the preserved antimicrobial activity of FR colostrum.

Immunoglobulins are highly abundant in bovine colostrum. Immunoglobulin G, for instance, is the predominant antibacterial protein in colostrum and acts by binding and neutralizing or opsonizing *E. coli* (Tortadès et al., 2023). However, IgG concentration is thought to be stable in CR and during HT and short-term FR (Rabaza et al., 2023; Westhoff and Mann, 2025), and thus is unlikely to underlie the observed differences in our study. Immunoglobulin A is the major mucosal Ig and acts similarly to IgG by binding and neutralizing bacteria (de Sousa-Pereira and Woof, 2019). Prior studies in our laboratory showed that HT decreased the concentration of IgA (Mann et al., 2020), whereas short-term FR maintained IgA concentrations (Westhoff and Mann, 2025). We have also shown that IgA and complement proteins were at their greatest concentration and activity level, respectively, in fresh compared with HT colostrum (Mann et al., 2020; Chandler et al., 2023). Moreover, calves fed CR have been shown to have lower serum concentrations of IgA, which could reflect lower IgA concentration (Godden et al., 2009). Therefore, IgA abundance might help explain the difference between CR, HT, and FR colostrum in its ability to control bacterial growth.

Low-abundance, but heat-labile proteins also contribute antibacterial properties; among these are lactoferrin, complement, and lysozyme (Hernandez et al., 1979). Lactoferrin, for example, is known to sequester iron and bind to the surface of bacteria (Visca et al., 1990). Specifically, it has been shown that iron-depleted, or apo-lactoferrin denatures near 60°C, while iron-saturated or holo-lactoferrin denatures at 90°C (Goulding et al., 2021). Interestingly, all forms of bovine lactoferrin are more heat-sensitive (i.e., denature faster) when heated in milk compared with phosphate buffer (Sánchez et al., 1992).

Another class of proteins in colostrum, complement proteins, directly kill pathogens via a pore-forming membrane attack complex or act indirectly through opsonization (Ricklin et al., 2010). Given the multiple antibacterial activities, inactivation of complement with heat as previously demonstrated (Chandler et al., 2023) likely underlies at least part of the decrease in antibacterial activity seen in HT, but not FR or RW treatment groups.

Lysozyme also kills directly by degrading the peptidoglycan cell wall of gram-positive bacteria, and acts indirectly by binding endotoxins, increasing IgA production, and activating macrophages (Sousa et al., 2014). Prior studies have demonstrated that HT via the LTLT method decreased lysozyme activity by up to 44% in human colostrum (Evans et al., 1978; Sousa et al., 2014). In contrast, storage at −20°C for 3 d to 3 mo did not affect the concentration of donkey or human milk lysozyme (Evans et al., 1978; Ozturkoglu-Budak, 2018). Although these studies have not been, to our knowledge, carried out with bovine lysozyme, it is plausible that denaturation of lysozyme due to HT, but not FR, contributed to the increased proliferation of *E. coli* in HT bovine colostrum compared with RW and FR counterparts in the current study.

We performed our work with a single bacterial strain of bovine fecal *E. coli* because this is a common contaminant, but our work should be extended to different bacterial isolates that can contaminate colostrum to better understand the breadth of colostrum's antimicrobial activity. Although antimicrobial peptides like complement and lactoferrin are nonspecific, Ig are antigen specific. Past studies with human breast milk and colostrum showed varied bactericidal activity against *Pseudomonas aeruginosa* (Takci et al., 2012) and across several different gram-negative and gram-positive species (Dolan et al., 1986), suggesting antimicrobial factors have varying efficacy against different bacteria.

A practical consideration that was not evaluated in this study was the duration of storage. In Westhoff and Mann (2025), freezing bovine colostrum at −20°C decreased IgG concentration by 32 wk, but did not affect IgM or IgA concentrations when stored for 1 yr. Additionally, bovine colostrum that had been previously heated via the LTLT protocol and then was frozen, showed decreased IgG and IgM concentrations when stored for 6 mo (Abd El-Fattah et al., 2014). Studies using human colostrum have also demonstrated decreased bactericidal activity against *E. coli* after 3 mo of freezing (Ogundele, 2002) and varying stability of bioactive factors including IgA, growth factors, and cytokines after up to 12 mo in the freezer (Ramírez-Santana et al., 2012). We acknowledge that our final sample size was below our intended sample size because the number of contaminated samples was larger than the anticipated 20%. However, the effect size observed in this study was also larger than anticipated, and statistical power was achieved with 7 samples.

An important implication of our results for colostrum management was that although HT may initially reduce pathogens (Wörmann et al., 2023), the resulting colostrum is likely more susceptible to bacterial growth upon subsequent or due to remaining contamination. This highlights a critical need for HT colostrum to be handled hygienically at all steps between harvest and feeding.
